# Experiences With an In-Bed Real-Time Motion Monitoring System on a Geriatric Ward: Mixed Methods Study

**DOI:** 10.2196/63572

**Published:** 2025-03-04

**Authors:** Stefan Walzer, Isabel Schön, Johanna Pfeil, Nicola Merz, Helga Marx, Sven Ziegler, Christophe Kunze

**Affiliations:** 1 Care and Technology Lab Furtwangen University Furtwangen im Schwarzwald Germany; 2 AGP Social Research FIVE e.V. Freiburg Germany; 3 Center of Implementing Nursing Care Innovations Freiburg, Nursing Direction Medical Center University of Freiburg Freiburg Germany

**Keywords:** nurses, geriatric patients, cognitive impairment, technology, fall prevention, hospital, mixed methods, patient, learning process, assessment, autonomy, impairment, real-time motion, university, geriatric ward, survey, anxiety, willingness, patient privacy, effectiveness, monitoring system, health care practice

## Abstract

**Background:**

Older adults now make up about two-thirds of hospital admissions, with up to 50% experiencing cognitive impairments such as dementia. These patients often struggle with adherence to care plans and maintaining regular day or night cycles, presenting challenges for nurses. Hospitals are typically unprepared to manage this patient population, resulting in increased nurse workload and challenges like managing motor agitation, which can lead to falls or accidental removal of medical devices.

**Objective:**

This study aimed to (1) assess how an in-bed real-time motion monitoring system (IRMS) impacts nurses’ perceptions of physical and mental stress, (2) evaluate the IRMS’s effect on the care process, (3) explore ethical implications like patient autonomy and privacy, and (4) understand how nurses acquire knowledge about the technology and how this affects their assessment of the IRMS.

**Methods:**

The IRMS, which provides real-time motion monitoring and bed edge or exit information, was implemented in the geriatric ward of a university medical center. The study followed a monocentric, explorative evaluation design using a mixed methods approach. It lasted 24 weeks and had two phases. In Phase 0 (6 weeks), patients received standard care. In Phase 1 (18 weeks), the IRMS was introduced. Initial data were gathered through focus groups and participant observations during manufacturer training sessions. At the end of the intervention, a survey, a second focus group, and an interview were conducted to capture nurses’ experiences. The study follows the Good Reporting of a Mixed Method Study (GRAMMS) checklist for reporting.

**Results:**

Initial training sessions with 12 participants (10 nurses and 2 physiotherapists) showed varying levels of engagement, with the second session demonstrating more optimism and interprofessional collaboration. A total of 10 questionnaires were completed (10/21, 48%). Survey results showed that 80% (8/10) of nurses found the IRMS valuable for assessing the quality of work, and 90% (9/10) were willing to continue using it. The system was regarded as reliable for monitoring bed edge and exit events. Usability was positively rated, with minimal concerns about documentation burden. Focus group discussions (n=3 per session) indicated that nurses viewed the system as reliable and appreciated its role in reducing anxiety related to fall prevention. However, concerns about patient privacy and monitoring were raised. Nurses expressed a willingness to continue using the IRMS but reaffirmed their ability to care for patients without it.

**Conclusions:**

Nurses had a generally positive attitude toward the IRMS, recognizing its benefits, particularly for nighttime monitoring. Although its effectiveness in preventing falls remains inconclusive, the system helps reduce nurses’ fear of falls and enhances their responsiveness. The study highlights the broader impact of the IRMS beyond fall prevention and stresses the importance of thoughtful integration into health care practice.

## Introduction

### Background

The global increase in emergency hospital admissions of older individuals is expected to continue, driven by ongoing demographic shifts. Currently, older adults make up approximately two-thirds of hospital inpatients, with up to 50% of this population experiencing some degree of cognitive impairment, including dementia-related conditions [1-3]. Patients with cognitive impairment (hereafter referred to as patients) often struggle to adhere to care plans in the hospital setting, and their day or night cycles are frequently disrupted. This presents additional challenges for nurses, as their workload increases, especially since hospitals are often not adequately equipped to care for patients with cognitive impairment [4]. A particular challenge for hospital nurses is motor agitation in and around the patient’s bed, which can lead to incidents such as falls or accidental removal of catheters or vascular access devices [5]. In light of these challenges, there is ongoing discussion about the potential role of technological innovations in supporting nurses and caregivers [6,7].

Bed exit information systems (BES) are well-known in this context [8,9]. In a previous study by the research team [10], the focus was widened to the complex interplay of factors from the nurses’ perspective. The study addressed the perception of nurses on several aspects as adverse events and the implementation in the clinical setting, as well as their assessment of usefulness. Therefore, nurses on regular wards at a university medical center used a bed exit system to better understand its potential in hospitals [10]. The system, which was placed under the mattress in the patient’s bed, informed nurses via the nurse call system whether patients were mobilizing to the edge of the bed or leaving the bed unassisted. An advantage of this system was its ease of use, which provided benefits to the nursing staff. However, due to the complex and sometimes unpredictable course of symptoms, it was only possible to use it in a targeted way for a limited number of patients (patients with cognitive impairments who also have a tendency to walk and a risk of falls or may become disoriented). That technological limitations and special characteristics strongly affect the effect of a BES becomes also evident in the study of Considine et al [11], in which alarms at the bedside caused specific challenges for the nurses, including issues related to alarm fatigue, disruptions in patient care, and difficulties in prioritizing responses to alarms.

Therefore, the research team looked for another technology with a higher degree of functionality (allowing for the setting of either bed edge or bed exit notifications) and an additional function (real-time motion monitoring), with the aim of evaluating its implementation. Here as well, the research question focused on possible benefits for the nurses in patient care and the integration of the technology in the working routines. To answer this question, the nursing staff of a geriatric ward in a university hospital used the Mobility Monitor, an in-bed real-time motion monitoring system (IRMS) from the company Compliant Concept AG, as an additional aid in the standard care of patients [12]. In addition to bed edge and bed exit information, the system provides nurses with real-time monitoring to observe and analyze the patient’s movement patterns in bed. In a previous evaluation project [13], the IRMS was found to have potential benefits for the nightly monitoring of patients on standard wards in general and the care of patients with delirium in particular.

Overall, previous studies have predominantly addressed single aspects of the technology, such as the efficacy and effectiveness of BES in terms of reducing falls or fall rates [14,15], and there has been limited research into nurses’ perspectives and experiences of using BES [16,17].

### Aim and Research Question

Guided by the results of previous studies, the purpose of this study was to better understand the positive effects and disadvantages of the use of an IRMS from a nursing point of view.

The study was designed to address the following research questions:

- How does the use of IRMS affect nurses’ perception of physical and mental stress?
- How is the care process affected by the use of the IRMS?
- What kind of ethical implications could arise from the nursing perspective, such as perceptions of autonomy and privacy for patients?
- How do nurses acquire knowledge about the technology, and how does this learning process influence their assessment of it?

## Methods

Reporting was guided by the Good Reporting of a Mixed Method Study (GRAMMS) criteria proposed by O’Cathain et al [18].

### Design

**Figure 1:**
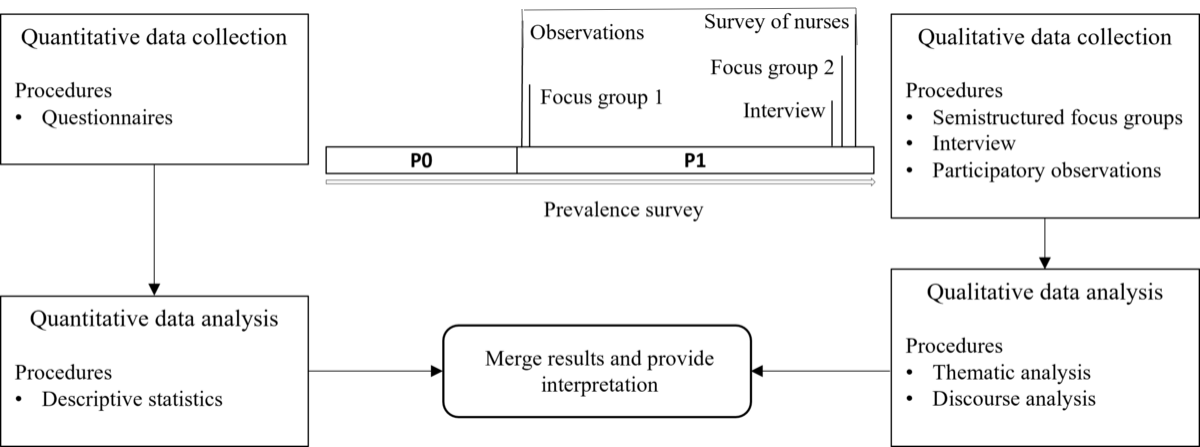
Parallel mixed methods triangulation study design.

This study was designed using a parallel mixed methods triangulation approach [19], incorporating a quantitative survey, qualitative focus groups, and one interview, as well as participant observations to obtain a more comprehensive understanding from multiple perspectives (Figure 1). A key principle of mixed methods research is that combining quantitative and qualitative data provides a richer understanding of the research question compared to using either data source in isolation [20]. The integration of results is realized in the Discussion section of this paper.

The study was conducted in a geriatric ward at a university medical center in the south of Germany. The ward has 20 beds with an average occupancy of 18 patients at any one time. Staffing typically includes three registered nurses on the day shift and usually three on the evening shift, but occasionally two registered nurses and two nursing assistants, depending on availability. During the night shift, there are usually two staff members on duty. Three physician assistants and one supervising physician are responsible for the ward.

Spanning 24 weeks, the study period was divided into two distinct phases. Phase 0 (baseline survey P0) lasted 6 weeks. During this phase, patients were cared for according to the university medical center care standard. Phase 1 (intervention phase P1) lasted 18 weeks. For this intervention phase, the geriatric ward was equipped with the IRMS, and data about the experience of nurses were collected. The IRMS were available to nurses as an aid to patient care, assisting nurses in assessing the patients’ cognitive status and tendency to get out of bed. Nurses were able to make their own decisions about which patients they would use the IRMS with. Both the (1) real-time monitoring of the patient’s movement patterns in bed and (2) bed edge and bed exit information could be turned on or off by the nurses.

During the study, 22 devices were made available to the ward from the first day of the intervention phase (Phase 1), ensuring full coverage. The research team integrated the devices into the beds on that day, which also marked the first training session, with a second session conducted the following week. Preparatory work, including software installation and setup of the screens in the nursing station, was carried out in the weeks prior (Phase 0). The devices were customized by the supplier (Compliant Concept) in coordination with the University Medical Center’s technical team to ensure compatibility with the call system.

The training consisted of a 2-hour session, which was held twice by a company representative for the nursing staff. While the focus was primarily on nurses, other professionals working on the ward, such as physicians and physiotherapists, were also invited to participate. The training included a presentation for the IRMS introduction, as well as practical exercises to reinforce learning. Additionally, research team members provided further assistance as needed, ensuring ongoing support for any questions or challenges the nursing team faced. Informational materials were also available to the staff, including a PowerPoint (Microsoft Corp) presentation, a practical written guideline for patient entry into the software, and the company’s manual. Regarding communication with patients about the IRMS, nurses were not specifically trained to discuss its use, but it ultimately depended on their professionalism in interacting with patients.

### Participants

The survey and focus group sessions involved nurses who met the predetermined criteria outlined in Textbox 1. A total of 21 nurses met the inclusion criteria at P1. Potential participants were informed about the survey, focus groups, participant observations, and interview through ward meetings and internal institutional mailing lists, supplemented by email communications. They were then formally invited to participate. Nurses who expressed an interest were given full written information about the study and the nature of their participation. The survey, focus groups, participant observation, and interview were conducted during working hours with the authorization of the respective organization and no additional incentives or rewards were provided to participants.

**Inclusion and exclusion criteria.**

**Inclusion criteria**

- ≥18 years
- Nurses with at least three years of apprenticeship or equivalent international training with professional recognition in Germany
- Employees working on the included ward during the study period

**Exclusion criteria**

- Employees of the included ward who belong to other professional groups.
- Employees of the reserve pool.
- Nurses from other wards helping out
- Trainees

### Data Collection

The study followed a parallel mixed methods approach with the following elements (see Figure 1 for an overview of the study design):

- Survey of nurses at time point P1.
- Focus groups with nurses at time points P0 and P1, before and after IRMS use.
- Interview with one nurse at time point P1, after IRMS use.
- Participant observations during the initial introduction of the technology on the ward by the manufacturer.

### Nurse Survey

The survey was developed in REDCap (Research Electronic Data Capture; Vanderbilt University) [21]. A comprehensive review of the literature did not identify a suitable assessment tool for collecting data on this topic in the German language. The web-based survey, which consisted of 48 items, was based on the technology attitude or acceptance, burden, and expectations or experiences survey used in a previous study [10]. It primarily used items from Spagnolli et al’s [22] survey on user acceptance of wearable technologies, which were translated into German by our team, and a subset of Isfort et al’s [23] survey on nurses’ perceived burden in patient care, which was available in German. The instrument was further supplemented with custom questions and sociodemographic data, including age, gender, and years of professional experience of the nursing staff. However, due to the small sample size, these sociodemographic data were deemed insufficient for meaningful subgroup analysis or generalizable insights. Presenting these variables could risk overinterpretation or imply trends unsupported by the data. Therefore, we chose to focus on the main findings directly related to our research questions. In previous projects using paper and pencil surveys, it was found that the nursing team preferred a digital format due to its environmental benefits, such as the elimination of paper waste. A total of 21 nurses were invited to participate via email with a link to the web-based survey, allowing them to complete the survey at their convenience. The survey began immediately after the intervention phase, starting 11 days after the IRMS had been in use for 20 weeks. The survey is included in Multimedia Appendix 1.

### Focus Groups and Interview

Each focus group was conducted during the first survey phase (P0) and at the end of the second survey phase (P1) by two researchers (IS and SW) to capture the context of technology use and to further explore the issues identified in the staff survey. The initial focus group guide addressed expectations regarding the potential for alleviation, usability, and the influence on social interaction with patients (Multimedia Appendix 2). In contrast, the guide for the discussion after the intervention centered on the experiences related to these same themes. Topics included the challenges of caring for patients with cognitive impairment, the tendency of patients to get out of bed unsupervised, and the expectations, experiences, and evaluations related to the use of the IRMS (Multimedia Appendix 3). This approach aimed to derive insights into nurses’ perceptions of physical and mental stress, as well as any changes resulting from the implementation of the technology. Inferences about potential alterations in the care process, patient outcomes, and the perceptions of autonomy or privacy by patients can be deduced from nurses’ comments on processes, perceptions, and evaluations before, during, and after the use of the IRMS.

The two researchers facilitating the discussions both have prior experience in conducting focus groups, with one having a background in nursing science (SW) and the other in sociology (IS). A semistructured interview guide was used, focusing on the concepts of interest while allowing for flexibility. The structure and content of the guide were informed by the core findings of previous studies [10,13] and the literature reviewed (see Background under Introduction section). Since one participant from the first focus group could not participate in the second, a follow-up interview was conducted using the same guideline at P1. Both the focus group and the interview were audio-recorded and transcribed using a denaturalized approach, omitting idiosyncratic speech elements [24]. Transcripts were pseudonymized prior to analysis.

### Participant Observations

The analysis of the focus groups in a previous study [10] showed that the initial introduction of the technology on the ward by the manufacturer is an important factor for the further course of implementation. Therefore, this factor was investigated as a decisive condition for the implementation of IRMS. For this purpose, observations in the sense of a focused ethnography [25] were carried out by two researchers (SW and IS) during the training sessions. The observations were open with the consent of the participants and the trainers. Field notes taken during the observations were collected, converted to a digital text format, and pseudonymized prior to analysis.

### Analysis

Qualitative data (focus groups, participant observation, and interview) were analyzed by SW, IS, and NM. We systematically organized and structured the data according to the principles of Kuckartz’s content analysis approach [26]. The main categories were shaped by the guiding questions and supplemented with inductive aspects derived from analysis discussions. Additionally, the investigation of specific aspects of the discussion dynamics in the focus groups was inspired by elements of the documentary method, particularly as outlined by Bohnsack [27] and Bohnsack and Schäffer [28]. This methodological combination aimed to provide a robust foundation for analyzing both the explicit content structure and the underlying social dynamics and interpretations of meaning, thereby promoting a comprehensive understanding of the phenomena under study. Descriptive quantitative data analysis by SW, IS, and HM was performed using SPSS Statistics (IBM Corp) for Windows. Frequencies and percentages were calculated for all quantitative variables.

### Setting

The study was conducted at the University Medical Center Freiburg.

### Ethical Considerations

The conceptualization and implementation of this study were based on the principles of the Declaration of Helsinki. By German law, survey studies with a focus on employees must be approved by the Employee Council at the respective institution. The responsible committee of the Employee Council of the University Medical Center Freiburg provided approval for this study in written format (approval 106/20 [MPG §23b]). Participation in the focus groups was voluntary, no personal data were collected, and anonymity was always maintained. All potential participants received written information on the study (reason for the study, objective, processes, and data protection), were informed about the decision of the Employee Council and its subcommittees, and had the opportunity to contact the investigators in case of questions at any time during the study. Informed consent to participate was assumed if individuals completed the survey and were confirmed (by ticking a box) at the beginning of the survey.

## Results

### Participant Observations

The two initial training sessions were diverging. There were 12 participants in total—10 nurses and 2 physiotherapists. They took place in two very different places. The first one was in a lecture hall where the participants were spread around the room. They seemed quite tired after the morning shift but were eager to understand the technology. The amount of information seemed rather overwhelming in this setting. However, the nurses seemed to be able to follow very well. The second introduction took place on the ward with two participants from the physiotherapy profession. The atmosphere was livelier and more optimistic regarding the use of the technology. There was an interprofessional exchange, and strategies for using the IRMS were discussed. Once again, the participants’ questions demonstrated their engagement and ability to quickly grasp the basic concepts of the technology. The presenter began the session by mentioning the number of miles nurses walk every day. However, this topic was not explored further in the discussion, and we conclude that the participants did not prioritize it as a goal for using the technology. Additionally, the presenter emphasized the expertise of the nurses in making decisions about the use of the IRMS. The presentation of case scenarios primarily focused on night-time use. Although preventing falls and other adverse events was framed as a key interest of the research team, the representative highlighted both fall prevention and motion analysis, with a particular focus on the latter. One participant’s question suggested that targeting specific patients might be a promising strategy.

### Survey

At the time of the survey, participants had been using the IRMS on the ward for 20 weeks and had gained initial experience of its functionality. A total of 11 of a possible 21 individuals participated in the survey, 10 of which were completed (10/21, 48%). Nurses’ responses to the survey confirmed that caring for patients with cognitive impairment is a significant burden (items 8-19 in Multimedia Appendix 1). Notably, participants demonstrated a general openness and minimal fear of technology (items 1-7 in Multimedia Appendix 1).

**Figure 2:**
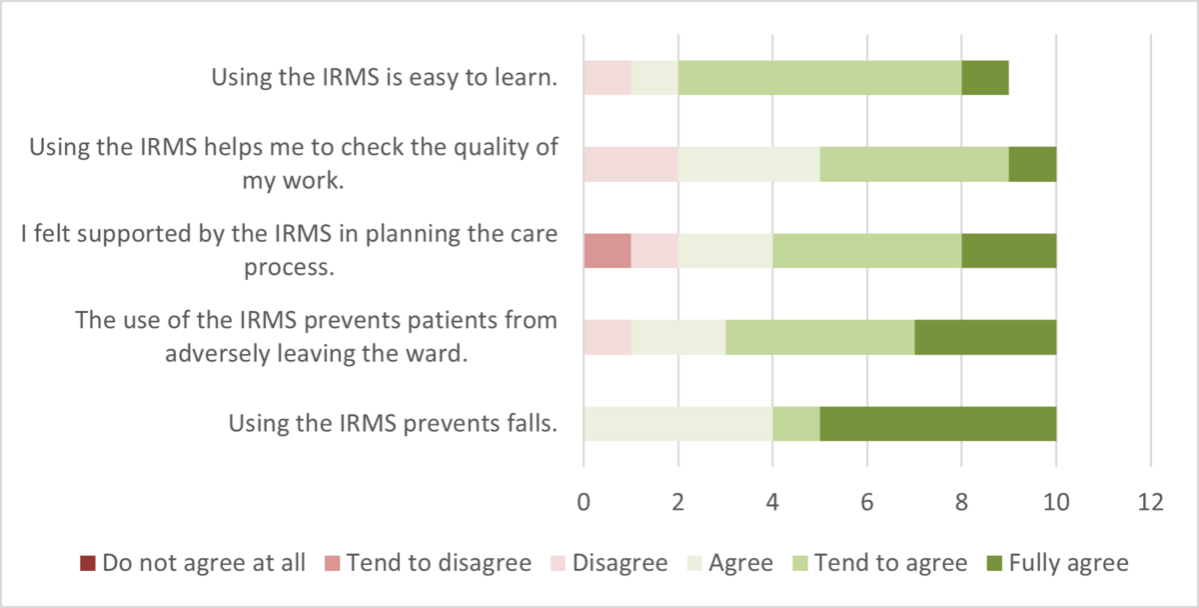
Survey results on the effectiveness and organizational aspects of the in-bed real-time motion monitoring system (IRMS).

When asked about potential improvements from the IRMS, most respondents were optimistic and agreed that it would improve outcomes in several areas. The only areas where they did not agree were preventing the removal of peripheral venous catheters and drains, improving privacy, and disturbing other patients (items 23, 25, and 26 in Multimedia Appendix 1). However, nurses also see many opportunities to improve care and work processes through the use of technology, including feeling safer due to the use of the IRMS, receiving support in care planning, and assisting in coordinating their work schedule (items 28-30 in Multimedia Appendix 1). The impact on patient safety is also seen as positive (item 20 in Multimedia Appendix 1). The survey responses from the nurses indicate a clearly positive view regarding the IRMS’s effectiveness in preventing falls and preventing patients from leaving the ward unnoticed (Figure 2).

In terms of organizational aspects, nurses found the IRMS to be a valuable tool for care planning and time management (Figure 2). In addition, 80% (n=8) of respondents (statements from “tend to agree” to “fully agree”) confirmed that the technology enabled them to assess the quality of their work (Figure 2). For nurses to find the IRMS helpful, they must find it reliable—which they consistently confirmed in the survey, both for bed edge and bed exit information and for motion monitoring (items 40-42 in Multimedia Appendix 1). In terms of usability, respondents generally agreed that using the technology was not annoying and did not increase the documentation burden. However, the initial setup was considered by some to be labor-intensive. Opinions varied between respondents, possibly influenced by the actual involvement in the setup process. (items 32, 33, and 37 in Multimedia Appendix 1). Overall, nurses found it easy to learn how to use the device (Figure 2).

Responses regarding the potential reduction of work-related movements, such as checking on patients at risk of falling, were mixed, with differing opinions between participants. Further insight into this variation can be gained by analyzing the focus groups, as described below.

Ethical considerations were explored by assessing whether nurses felt that the IRMS helped to maintain patient privacy. In general, respondents tended to think that it did not, a nuance that may be clarified by analysis of the focus groups. When asked if they felt monitored by the technology, 8 out of 10 nurses said that this was not the case. Almost all of the nurses surveyed (n=9; statements from agree to fully agree) indicated that they would continue to use the IRMS in the future.

### Focus Groups and Interview

#### Overview

The focus groups and the interview largely confirm the results of the survey and can shed more light on some details. Each focus group had a total of three participants, with one person attending both sessions. The issues of benefits, privacy concerns, and usability were discussed more controversially than the survey suggests. This is illustrated by the following analyses. A summary of the themes, including their descriptions and primary insights, is provided in Table 1. The originals of the quotes used can be found in Multimedia Appendix 4.

**Table 1:** Summary of themes and key insights from focus groups and the interview.

Theme	Description	Key insights
Benefits	Discussion on the benefits of the IRMS^a^, particularly regarding fall prevention and night shift management.	The IRMS was seen as helpful in improving patient safety and reducing anxiety through quick responses. It was especially valuable during night shifts to manage patient movements.
Privacy concerns	Concerns regarding patient privacy in relation to monitoring through the IRMS.	Some patients felt monitored or “controlled.” However, this was considered an exception. A clear explanation of the system helped alleviate some concerns.
Usability	Experiences of nurses with the usability and reliability of the system.	The IRMS was considered increasingly reliable. Suggestions for improvement mainly focused on integration with the documentation system and reducing “false alarms.”
Overall assessment	Overall assessment of the system and nurses’ willingness to continue using the IRMS.	Nurses found the system helpful, but not essential. They emphasized that care could still be provided without it, though some additional visual checks would be necessary.

^a^IRMS: in-bed real-time motion monitoring system.

#### Benefits

Nurses participating in both focus groups reported that they found the technology helpful in preventing or responding to falls. However, reports of specific responses to information about bed exit or bed edge information remained largely vague despite repeated questioning. The meaning of the “usefulness” of the IRMS becomes clearer when considering that the additional information allows nurses to better plan their next steps, potentially allowing them to respond more quickly to falls.

And when it rings, we know, this woman is sitting at the edge of the bed. Then we know...We have ideas. Then we know how we have to organize the process or how to do the plan for the patient.

Focus Group 1, P3

In addition, the absence of narratives about specific fall-prevention situations emphasizes that it was not the prevention of falls themselves that played a crucial and relieving role, but rather a sense of safety in relation to falls. Simply knowing that they would be alerted if the patient got up allowed nurses to perform their other activities with less anxiety.

Well. Especially regarding falls there was a certain safety, because the notification actually always sets off and you don’t have to permanently do visual controls and go to the rooms. This certainly creates a feeling of safety, to know “Okay, the patient is not in danger of falls or similar right now.” Well and for coverage of the positioning and in order to see: “Okay, was there sufficient positioning? Have I supported the patient sufficiently?...it gives a certain feeling of safety when you can check this on the monitor.”

Focus Group 2, P3

In addition to an increased sense of security, another clearly identified benefit is improved information about patient self-movement. This is particularly important at night, when patients may need to be repositioned at defined intervals according to nursing standards. Right at the beginning of the interview, the IRMS is described as a technology that goes beyond and is superior to the existing technical possibilities (eg, a mat on the floor in front of the bed that sends a signal to the call system when touched by the patient) and is superior to them. According to the interviewee, the technology is particularly valuable during night shifts because it constantly monitors whether patients are moving or not. Previously, patients had to be awakened and repositioned every 2 to 3 hours to relieve pressure.

In nightshifts [with the IRMS] you immediately notice: Do people move, don’t they move. And before, okay you walk there every 2 to 3 hours, turn the people from left to right, have to wake them up and this is bad, when people are fast asleep and you see: Oh, he’s moving, moving sufficiently, perfect. AND, it’s very good that when somebody really has developed a decubitus here, we can prove: Okay, every 2 hours, the monitor has recorded it and there were immediate reactions, there were positionings and so we can demonstrate this.

Interview

The IRMS provides an indication of whether there has been sufficient spontaneous movement, allowing nurses to avoid unnecessary repositioning. This helps prevent unnecessary disruption to the patient’s sleep and supports nurses in re-evaluating their own judgments about the need for repositioning. If a patient develops a pressure ulcer, the recorded data can be used by nursing staff to demonstrate that regular pressure-relieving positioning has been carried out correctly (interview and focus groups). The interviewee also mentioned the recording of bed exits, especially for patients with hyperdelirium. The ability to detect physical tension in patients through specific movement patterns is highlighted as another, initially unintended, “function” of the device.

#### Privacy Concerns

The issue of privacy was not directly raised in the focus group discussions. However, the analysis provides insights into the different responses in the survey. The difference may be due to different perceptions or reference points of “privacy.” In the second focus group discussion, it was reported that with the use of the IRMS, room doors are occasionally closed more often, as monitoring can take place without direct visual contact. However, according to nurses’ reports, some patients occasionally expressed feeling monitored and controlled. For example, the interviewee reported a situation where the technology was seen by a patient as a “spy in the bed.”

This one patient, who always said: “Ha, the spy betrayed me again, actually I wanted to try to go to the toilet by myself.” There you stand next to them and say: “You shouldn’t walk alone; you are still quite weak-kneed with the rollator.”

Interview

Due to the design of the study, it remains uncertain how patients experience these reactions, whether positively or negatively. It is worth noting that the term “spying” typically carries a negative connotation in German. This anecdote, involving a patient at risk of falling who was attempting to use the toilet independently, illustrates the effectiveness of the IRMS in preventing potentially dangerous situations. The system alerted the nursing staff, enabling them to assist the patient immediately. The nurses perceive their immediate presence when the system is activated as a positive aspect, as it allows for a quick response to the patient’s needs. However, the extent to which patients are comfortable with this form of monitoring seems to be a critical factor.

P3: I think they were not really aware, that there is a special mat in their bed. They didn’t really notice it, most of them. The others quickly mentioned it. But they didn’t mind either, whether there was something like that or not. So.

P1: Except for the ones who ranted, because they were worried, well...one once even said: “Am I being controlled?”

P3: Okay no, I’ve never come across that.

P1: And then there were some, who worried [about] the space for their cell phone plug.

Focus Group 2, P1 and P3

This highlights an interesting ethical conflict. The nurses in the focus group at P1 also report patients’ complaints or confusion about the sense of being controlled, but they regard these as special cases, similar to minor concerns like charging their mobile phones. However, the primary cause of confusion appears to be the patient’s general state of confusion. Explaining the technology seemed effective in addressing this confusion. However, the nurses did not report exploring deeper complaints, such as the feeling of being controlled or monitored. This assessment by the nurses regarding patient privacy may correlate with their own perceptions of control associated with IRMS. In the first focus group at P0 and the interview (P1), some team members initially viewed the IRMS as a potential “control device,” referring to the supervision of nurses and their activities, for example, by line managers. Although this perception dissipated relatively quickly as the IRMS became more established, it highlights nurses’ concerns about potential surveillance, which were also discussed in the focus groups. After a longer period of use (according to reports from the second focus group), nurses no longer feared being monitored themselves. While in the first focus group, they were suspicious of the potential monitoring of their work performance, in the second focus group, they expressed hope that the IRMS would demonstrate that they were making correct judgments about patient positioning. The issue of surveillance was eventually dismissed as irrelevant.

#### Usability

With minor reservations, the nurses consider the system to be reliable, especially in the second focus group and the interview at P1, more so than at the beginning. They have no fundamental objections to the usability of the system, but they do have some suggestions for improvement. Most of these suggestions focus on improving the documentation process. For example, if there were an interface to the documentation system and a simplified or automated transfer of recorded data to mandatory documentation, the IRMS could streamline the documentation process overall. This could also help nurses to provide a plausible explanation for justified deviations from standards of care, thereby encouraging the use of their own professional knowledge and experience.

In addition to the survey’s assessment that the technology is easy to learn, focus group participants and the interviewee emphasize that its use requires the development of routines. They point out that a lack of familiarity with the system can lead to disruptions in ward operations, particularly among other professional groups who do not work exclusively on the participating ward and have not developed routines with the IRMS. This often results in “false alerts” during the day, such as physiotherapists forgetting to turn off alerts before their interventions.

In line with the inconsistent assessment in the survey regarding the reduction of walking distances, different perspectives emerged in the focus group discussions. When using the IRMS, nurses do not have to repeatedly check on patients at risk of falling by visiting them. This is especially an improvement at night. However, this process rarely exists in isolation, as nurses often combine these checks with other tasks. The implementation of the system does not alter these interrelated activities, which continue as usual.

Those who are hyperactive at night, you pick that up anyways, because then you are continuously close to the bed, because they’re ringing all the time, calling or doing anything else.

Focus Group 2, P1

#### Overall Assessment

When asked whether they would like to continue using the technology, the nurses in the second focus group were slightly more ambivalent than in the survey. On the one hand, they emphasize that care can be provided without such technology, but on the other hand, they generally find the IRMS helpful.

Moderator: And if there was no IRMS starting from tomorrow on. How would you like that?

Person 3: Well...

Several: [laughing]

Person 2: I would miss it. I would like to

Person 3: Yes, it would be missed. But the work still could be done. That’s not the way it is.

Person 2: Well maybe a thing or two would be more complicated again. Because we would have to do more visual controls at the patients’ beds, for patients who could wander or patients with dementia.

Person 3: Yes.

Person 1: Exactly, probably one fall or two would happen

2.5 seconds

Person 3: but those could not be prevented with IRMS, either.

Person 1: Yes, sure! We would certainly go back to text-book, which means positioning according to the clock, but-

Person 2: This would not make our work impossible

Person 1: (laughing) Exactly. Nursing can do that

Focus Group 2, P1, P2, and P3

Overall, the nurses expressed a willingness to continue using the IRMS but highlighted that care is not entirely dependent on such a technology. In the following discussion, possible backgrounds for this evaluation will be examined further.

## Discussion

### Principal Findings

The two initial training sessions showed differences in both effectiveness and atmosphere. The second introductory session, held on the ward with additional therapeutic staff, fostered a much livelier and more optimistic atmosphere toward the technology. Interprofessional exchange was seamless, with active discussion of strategies for implementation and use of the IRMS. These observations are consistent with the research of Koukourikos et al [29], which emphasizes the effectiveness of hands-on training in familiar environments over formal settings. Moreover, Kahn et al [30] highlight the importance of interprofessional collaboration from the outset of technology implementation in hospital care settings. Building on these findings, a recent study using a design-based research approach highlights the importance of structured training and support programs to help nurses effectively integrate innovative technologies into practice [31].

The survey, focus groups, and the interview showed that nurses benefit most from motion monitoring, especially at night. The increased sense of security can reduce psychological distress. This finding is consistent with the systematic review by Mileski et al [32], which included 28 studies. Whether the technology is effective in preventing falls could not be determined as this study was not designed to measure its effectiveness and the nurses’ statements in this regard remain ambiguous. However, the technology does appear to reduce the nurses’ fear of falls and make them feel better equipped to respond in a timely and appropriate manner should a fall occur.

Interestingly, the focus on fall prevention was primarily driven by the research question, but it emerged that other benefits were more relevant. One reason for the limited focus on fall prevention might be that nurses’ responses to the information provided by the system were constrained by existing routines. Since there was no significant adaptation of processes beyond the introduction of the technology, nurses could not change their workflows significantly. More comprehensive changes—such as ongoing training programs and organizational policies supporting flexibility in nursing practices—would be needed for nurses to react more promptly when desired. Future studies would benefit from exploring how such ongoing support influences technology adoption and the potential long-term impact on nursing workflows.

The literature also suggests that alarms, particularly when they are frequent or audible to patients, may not be effective as standalone interventions [33-35]. A high number of alerts or alarms (especially if patients can hear them) may even have adverse effects, as suggested by the study by Considine et al [11]. Excessive alarms can increase anxiety and stress levels, disturb sleep, and desensitize both patients and staff, leading to delayed responses to critical situations. This may explain why the system was primarily used at night when nurses could respond more directly to the system’s alerts. Goals such as reducing physical strain by reducing the need to walk to check on patients are negated because such rounds often include other essential nursing activities. For example, during these rounds, nurses are often checking on multiple patients, assessing their comfort, administering medication, or performing other routine tasks. This underlines that technology can only reach its full potential if it is integrated into comprehensive, targeted changes and is part of a broader, well-structured care plan [32].

Another factor contributing to the mixed evaluation of the IRMS could be debates about the professionalization of nursing in Germany [36]. Notably, in the second focus group, it was emphasized that nursing care could still be provided effectively without the use of technology.

Concerns about surveillance disappeared over time. In the survey and the second focus group, nurses no longer expressed privacy concerns and even minimized patients’ worries. These findings are consistent with a previous study on a BES used across several wards at the University Medical Center [10]. On one hand, it could be argued that patient privacy is actually enhanced compared to personal observation, as physical movements can be monitored discreetly, without patients’ awareness. However, patients still voiced concerns about being monitored. While the focus group participants did not consider this a serious issue, it remains essential to address patient privacy. Nurses could explore ways to make the technology more understandable, ensuring that patients are informed and that their consent is respected. Situations may arise where a patient’s rejection of the technology—due to significant stress or a violation of personal values—should lead to discontinuation of the system’s use. Future research could explore effective communication strategies to address privacy concerns and promote patient acceptance of monitoring technologies.

Finally, integration with documentation standards and systems remains a major concern contributing to nurses’ workload, as discussed in the reviews by Mileski et al [32] and in studies on the implementation of digital nursing technologies [37-39]. Considering these aspects in the development of new technologies could significantly enhance the benefits of nursing. By supporting conditions that allow nurses to work in a self-directed and knowledge-based way, both their empowerment and professional standing could be strengthened.

### Limitations

When discussing the use of the IRMS, it is important to clarify that the study’s aim was not to assess its effectiveness or similar outcomes but rather to explore nurses’ perceptions of its usefulness.

One limitation of the study is the small sample size. Ideally, multiple focus group discussions would have been conducted and analyzed at each time point to identify specific discursive patterns through comparison. Unfortunately, this was not feasible due to a limited number of eligible participants and a lack of volunteers. For practical reasons, an interview was conducted instead of a focus group. While an interview does not capture the same level of social consensus on issues as a focus group, the results were notably similar. The survey achieved a response rate representing 48% of the nursing staff in the geriatric ward. Although the sample size was relatively small, it provided valuable insights into the perceptions of nearly half of the relevant nursing staff. Given the limited number of participants, it is possible that the findings could be influenced by selection bias, especially if the individuals who participated were more likely to be early adopters or held leadership positions within their wards. Early adopters, including those in leadership roles, may exhibit a more positive perception of new technologies, potentially leading to an overrepresentation of optimistic perspectives in our results.

However, it is important to note that this study does not aim to present representative findings. Instead, it seeks to provide an in-depth insight into the implementation and use of the technology within a specific context. In qualitative research, some degree of bias is inherent, as findings are inevitably tied to the specific social situations and perspectives of the participants involved.

### Conclusions

Based on the findings, the participating nurses have a positive attitude toward the use of a real-time in-bed movement monitoring system, highlighting its benefits for nurses and patients, particularly during nighttime monitoring. While its effectiveness in preventing falls remains inconclusive, the system appears to reduce nurses’ fear of falls and improve their ability to respond effectively. The results highlight the wider impact of the system beyond fall prevention and underline the importance of carefully integrating such technologies into health care practice. The study acknowledges the challenges of embedding technology into care, including the limitations of nurses’ responses to the IRMS information and ongoing discussions about professional dynamics in Germany. It also identifies opportunities for future research and development, particularly in relation to integration with documentation systems. Overall, the study argues for a deliberate, holistic approach to health care technology that considers professional relationships, patient needs, and system improvements.

## Appendix

Multimedia Appendix 1 Survey of the nursing staff.

Multimedia Appendix 2 Focus group guide I.

Multimedia Appendix 3 Focus group guide II.

Multimedia Appendix 4 Translated quotes.

## Abbreviations

BES: bed exit information system
GRAMMS: Good Reporting of a Mixed Method Study
IRMS: in-bed real-time motion monitoring system
REDCap: Research Electronic Data Capture

## Data Availability

The datasets generated and analyzed during this study are available from the corresponding author upon reasonable request.
