# Supplementary material for: Experiences With an In-Bed Real-Time Motion Monitoring System on a Geriatric Ward: Mixed Methods Study
Source: JMIR Form Res. 2025 Mar 4;9:e63572. doi: 10.2196/63572 (PMC11920652; doi:10.2196/63572)
Supplement: Multimedia Appendix 1 [file formative_v9i1e63572_app1.docx]

| Question block 1: In the following, we would like to learn more about your general attitude towards IT (information technology). | | |
| --- | --- | --- |
| **No.** | **Question** | **Matrix** |
| 1 | Overall, I think IT offers some advantages. | Don't agree at all  Disagree  Agree less  Tend to agree  Agree  Totally agree  I don't know |
| 2 | I think IT is indispensable today. | Don't agree at all  Disagree  Agree less  Tend to agree  Agree  Totally agree  I don't know |
| 3 | If I have to use IT, I'm afraid of breaking something or making irreversible mistakes. | Don't agree at all  Disagree  Agree less  Tend to agree  Agree  Totally agree  I don't know |
| 4 | Most IT-related questions are difficult for me. | Don't agree at all  Disagree  Agree less  Tend to agree  Agree  Totally agree  I don't know |
| 5 | The possibility of using IT that I have never used before scares me. | Don't agree at all  Disagree  Agree less  Tend to agree  Agree  Totally agree  I don't know |
| 6 | My experience with technology in general: | Slider (Min. 1, Max 10)  No experience, a lot of experience |
| 7 | My acceptance of technology in general: | Slider (Min. 1, Max 10)  No acceptance, high acceptance |
| Question block 2: In the following, we would like to find out how you assess your burden with regard to patients with cognitive impairments during the early shift. | | |
| 8 | The fact that patients with cognitive impairments could get out of bed unsupervised and fall weighs on me | very strong  strong  moderate  rather low  little  Not at all  I don't know |
| 9 | The fact that patients with cognitive impairments could leave the ward or their room unnoticed and get lost weighs on me | very strong  strong  moderate  rather low  little  Not at all  I don't know |
| 10 | The fact that I am tied to the patients with cognitive impairments in terms of time, but actually have to do other work, weighs on me | very strong  strong  moderate  rather low  little  Not at all  I don't know |
| 11 | The fact that I can't do justice to patients with cognitive impairments weighs on me | very strong  strong  moderate  rather low  little  Not at all  I don't know |
| Question block 3: In the following, we would like to find out how you assess your burden with regard to patients with cognitive impairments during the late shift. | | |
| 12 | The fact that patients with cognitive impairments could get out of bed unsupervised and fall weighs on me | very strong  strong  moderate  rather low  little  Not at all  I don't know |
| 13 | The fact that patients with cognitive impairments could leave the ward or their room unnoticed and get lost weighs on me | very strong  strong  moderate  rather low  little  Not at all  I don't know |
| 14 | The fact that I am tied to the patients with cognitive impairments in terms of time, but actually have to do other work, weighs on me | very strong  strong  moderate  rather low  little  Not at all  I don't know |
| 15 | The fact that I can't do justice to patients with cognitive impairments weighs on me | very strong  strong  moderate  rather low  little  Not at all  I don't know |
| Question block 4: In the following, we would like to find out how you assess your burden with regard to patients with cognitive impairments on night duty. | | |
| 16 | The fact that patients with cognitive impairments could get out of bed unsupervised and fall weighs on me | very strong  strong  moderate  rather low  little  Not at all  I don't know |
| 17 | The fact that patients with cognitive impairments could leave the ward or their room unnoticed and get lost weighs on me | very strong  strong  moderate  rather low  little  Not at all  I don't know |
| 18 | The fact that I am tied to the patients with cognitive impairments in terms of time, but actually have to do other work, weighs on me | very strong  strong  moderate  rather low  little  Not at all  I don't know |
| 19 | The fact that I can't do justice to patients with cognitive impairments weighs on me | very strong  strong  moderate  rather low  little  Not at all  I don't know |
| Question block 5: In the following, we would like to learn more about your experiences with the practical use of the Mobility Monitor. | | |
| 20 | In your opinion, how does the use of the Mobility Monitor affect patient safety? | Slider (Min. 1, Max. 10)  Negative, not at all, positive |
| 21 | In your opinion, how does the use of the Mobility Monitor affect the nursing relationship with patients? | Slider (Min. 1, Max. 10)  Negative, not at all, positive |
| 22 | The use of the Mobility Monitor prevents falls. | Don't agree at all  Disagree  Agree less  Tend to agree  Agree  Totally agree  I don't know |
| 23 | The use of the Mobility Monitor prevents the unwanted removal of drains and accesses. | Don't agree at all  Disagree  Agree less  Tend to agree  Agree  Totally agree  I don't know |
| 24 | The use of the Mobility Monitor prevents patients from leaving the ward unwanted. | Don't agree at all  Disagree  Agree less  Tend to agree  Agree  Totally agree  I don't know |
| 25 | The use of the Mobility Monitor prevents fellow patients from being disturbed by patients with cognitive impairments. | Don't agree at all  Disagree  Agree less  Tend to agree  Agree  Totally agree  I don't know |
| 26 | The use of the Mobility Monitor helps to protect the privacy of patients. | Don't agree at all  Disagree  Agree less  Tend to agree  Agree  Totally agree  I don't know |
| 27 | I was able to reduce my distances to the ward by using the Mobility Monitor. | Don't agree at all  Disagree  Agree less  Tend to agree  Agree  Totally agree  I don't know |
| 28 | I feel safer by using the Mobility Monitor. | Don't agree at all  Disagree  Agree less  Tend to agree  Agree  Totally agree  I don't know |
| 29 | I am supported in care planning by using the Mobility Monitor. | Don't agree at all  Disagree  Agree less  Tend to agree  Agree  Totally agree  I don't know |
| 30 | I feel supported in the temporal coordination of work by using the Mobility Monitor. | Don't agree at all  Disagree  Agree less  Tend to agree  Agree  Totally agree  I don't know |
| 31 | The use of the Mobility Monitor helps me to check the quality of my work myself. | Don't agree at all  Disagree  Agree less  Tend to agree  Agree  Totally agree  I don't know |
| 32 | The use of the Mobility Monitor is associated with increased documentation costs. | Don't agree at all  Disagree  Agree less  Tend to agree  Agree  Totally agree  I don't know |
| 33 | The effort required to set up the Mobility Monitor for patients is high. | Don't agree at all  Disagree  Agree less  Tend to agree  Agree  Totally agree  I don't know |
| 34 | I feel monitored by the Mobility Monitor. | Don't agree at all  Disagree  Agree less  Tend to agree  Agree  Totally agree  I don't know |
| 35 | How has your burden in the care of patients with cognitive impairments changed as a result of the use of the Mobility Monitor? | Slider (Min. 1, Max10)  greatly decreased, not at all, greatly increased |
| Question block 6: In the following, we would like to learn more about your attitude to the use of the Mobility Monitor. | | |
| 36 | The use of the Mobility Monitor is easy to learn. | Don't agree at all  Disagree  Agree less  Tend to agree  Agree  Totally agree  I don't know |
| 37 | Using the Mobility Monitor is annoying. | Don't agree at all  Disagree  Agree less  Tend to agree  Agree  Totally agree  I don't know |
| 38 | I can imagine continuing to use the Mobility Monitor in the future, beyond the project phase. | Don't agree at all  Disagree  Agree less  Tend to agree  Agree  Totally agree  I don't know |
| 39 | I feel well informed about the Mobility Monitor project. | Don't agree at all  Disagree  Agree less  Tend to agree  Agree  Totally agree  I don't know |
| 40 | The Mobility Monitor proved to be reliable in practical use with regard to its function of bed edge warning. | Don't agree at all  Disagree  Agree less  Tend to agree  Agree  Totally agree  I don't know |
| 41 | The Mobility Monitor proved to be reliable in practical use with regard to its function of bed exit warning. | Don't agree at all  Disagree  Agree less  Tend to agree  Agree  Totally agree  I don't know |
| 42 | The Mobility Monitor proved to be reliable in practical use with regard to its function of mobility monitoring (movement profile). | Don't agree at all  Disagree  Agree less  Tend to agree  Agree  Totally agree  I don't know |
| 43 | Were there any difficulties in the practical implementation during the project regarding the use of the Mobility Monitor?  If so, which ones: | Yes  no |
| 44 | Do you have any further suggestions or wishes regarding the use of the Mobility Monitor? |  |
| Sociodemographic information | | |
| 45 | Your age | up to 30 years  over 30 to 50 years  over 50 years  no information |
| 46 | Your gender | female  male  miscellaneous  an indication |
| 47 | How many years of professional experience do you have in health and nursing, health and pediatric nursing or geriatric care (without training period)? | up to 2 years  over 2 to 10 years  over 10 years  no information |
| 48 | How many years of professional experience in health and nursing, health and pediatric nursing or geriatric care (without training period) do you have in dealing with patients with cognitive impairments? | up to 2 years  over 2 to 10 years  over 10 years  no information |
