# Supplementary material for: Experiences With an In-Bed Real-Time Motion Monitoring System on a Geriatric Ward: Mixed Methods Study
Source: JMIR Form Res. 2025 Mar 4;9:e63572. doi: 10.2196/63572 (PMC11920652; doi:10.2196/63572)
Supplement: Multimedia Appendix 2 [file formative_v9i1e63572_app2.docx]

**Focus Group Interview I**

**Frame**

- Approximately 60 minutes
- 3-6 participants
- Guideline-based
- Implementation by 2 people (moderator + support/backup)
- Audio recording with round of introductions

**Organization / implementation of the focus group**

- A short introduction to the topic or a short reminder of the survey

**Focus Group Guide**

**Introduction:** Welcome and thank you for taking the time.

We are now conducting this group discussion to discuss with you your expectations with regard to the benefits and limitations of the Mobility Monitor and the necessary framework conditions for the use of assistive devices on your ward.

| **Topic area** |  | **Guiding question (narrative prompt)** | **Checklist** | **Note Demand** |
| --- | --- | --- | --- | --- |
| Expectations of the Mobility Monitor: Relief |  | If you are interested in strategies for dealing with patients with cogn. impairments. what do you expect, how could the Mobility Monitor help with this? | How and in what situations do you plan to use it?  Do the functions that the Mobility Monitor offers or that have just been presented to you seem useful for your tasks? | Hypothesis:  Technology is always perceived as something disruptive at first (reduction of social interactions) |
|  |  | Can you imagine situations where Mobility Monitor can be disruptive or stressful for you? | Keyword "alarm stress" |  |
|  |  | When you think of your pat. How will they react to Mobility Monitor? | Or the relatives? | Aspects that could be addressed: Sense of security vs. surveillance/restriction of autonomy, neglected need for care? |
| Expectations of the Mobility Monitor: Usability |  | How do you think the Mobility Monitor will be integrated into everyday care? | Will he fit into the processes?  Does it fit in with the care tasks?  What do you think could cause it to fail? |  |
|  |  | Do you think Mobility Monitor reliably performs the functions presented in the training? | e.g., data collection  Alarms set |  |
| Social interaction |  | In your opinion, does the use of Mobility Monitor have an influence on the interaction with patients? | How do you think the nature or density of encounters with patients could change? |  |
|  |  | Such aids are often used with the aim of minimizing distances to patients - what do you think of this/how do you see it? | What positive aspects can you imagine?  What negative sides could this have?  Possible impulses:  Do you think this could increase the privacy of the patients?  Do you think it is realistic that there will be more time for interpersonal interaction elsewhere due to 'saved' trips to the patients? | If necessary, refer to training |
|  |  | Is the Mobility Monitor a system you wanted? |  |  |

**End:**

Are there any other aspects of this topic that we have not yet addressed and that concern you?

**Thank you for the time and openness you took!**
