# Supplementary material for: Experiences With an In-Bed Real-Time Motion Monitoring System on a Geriatric Ward: Mixed Methods Study
Source: JMIR Form Res. 2025 Mar 4;9:e63572. doi: 10.2196/63572 (PMC11920652; doi:10.2196/63572)
Supplement: Multimedia Appendix 3 [file formative_v9i1e63572_app3.docx]

**Focus Group Interview II**

**Frame**

- Approximately 60 minutes
- 3-6 participants
- Guideline-based
- Implementation by 2 people (moderator + support/backup)
- Audio recording with round of introductions

**Organization / implementation of the focus group**

- A short introduction to the topic or a short reminder of the survey

**Focus Group Guide**

**Introduction:** Welcome and thank you very much for taking the time! A few months ago, there was already a discussion with colleagues of yours when the Mobility Monitor was just newly introduced on the ward. Now that you have had some experience with the Mobility Monitor, we would like to talk to you today about your experiences with the Mobility Monitor. What benefits do you see in it and where does the device reach its limits? We have again prepared a rough guide with different topics, and look forward to discussing it as openly and freely as possible. Anyone can say anything; or say nothing! Everything that is said here in this room will be treated completely confidentially and, if possible, anonymized.

| **Topic area** |  | **Guiding question (narrative prompt)** | **Checklist** | **Note Demand** |
| --- | --- | --- | --- | --- |
| Overarching decision on the benefits of the technology (role play) |  | Put yourself in the role of a ward manager. You have the option of purchasing doorbell mats or the Mobility Monitor (or neither). What would you choose? And why? | For what reasons do they decide for or against the hypothetical "purchase" of the Mobility Monitor? | If necessary, also bring the costs of the systems into the discussion, if it makes sense. |
| Experiences with the Mobility Monitor: Relief | 2 | Please describe a few concrete situations in which you have used Mobility Monitor. | Have concrete situations described  Tendency to go into the general 🡪 Have specific cases described in specific patients.  Bed edge info/exit info?  At night? | Hypothesis:  Technology is always perceived as something disruptive at first (reduction of social interactions)  At night, Mobility Monitor is given a higher benefit, as there is much more going on on the ward during the day. |
|  |  | For what reasons? So how did you make these decisions? |  |  |
|  |  | What did you do with the information that Mobility Monitor provides you?  How did you proceed with it? | Have concrete situations described  How did the Mobility Monitor relieve you at work? Temporal, psychological, etc.; … encumbered? | Hypothesis:  Ringing stress due to "false alarms" or incorrect settings.  More safety and fewer walking distances, bearings when used correctly/  Selection Objectives of the manufacturers:  Fewer "unnecessary" walking distances  Individual positioning intervals and thus better pressure ulcer prophylaxis, in some cases less "stress" for patients and caregivers  And thus more time for other tasks |
|  |  | How have your patients reacted to Mobility Monitor? | Or the relatives?  Have concrete situations described | Aspects that could be addressed: Sense of security vs. surveillance/restriction of autonomy, neglected need for care? |
| Experiences with the Mobility Monitor: Usability |  | How has Mobility Monitor integrated into everyday care? If you compare it to digital documentation, for example. | Does it fit in with the care tasks?  What are the reasons for or against?  Ask for specific situations to be described | Differentiation between hardware (preparation, calibration, etc.) and software (additional data) |
|  |  | What technical or organizational things work very well at Mobility Monitor and what doesn't work so well? | e.g., data collection  Alarms set  Ask for specific situations to be described | During the first discussion, various restrictions were mentioned:  - Screen too small  - Settings can only be changed at the bedside  - Not mobile  - Too inaccurate alarms (e.g., for hyperactive patients) |
| Social interaction |  | What influence does the use of Mobility Monitor have on the interaction with patients? | Are the ways patients are treated changing and if so, for what reasons?  What negative sides could this have for patients/care?  Possible impulses:  Do you think this could increase the privacy of the patients?  Do you think it is realistic that there will be more time for interpersonal interaction elsewhere due to 'saved' trips to the patients? | Hypothesis:  Little/hardly any influence, because no less attention is paid to the patients and |
|  |  | How would you like it if the Mobility Monitor was no longer on the ward tomorrow? |  |  |

**End:**

Are there any other aspects of this topic that we have not yet addressed and that concern you?

**Thank you for the time and openness you took!**
