# Supplementary material for: Experiences With an In-Bed Real-Time Motion Monitoring System on a Geriatric Ward: Mixed Methods Study
Source: JMIR Form Res. 2025 Mar 4;9:e63572. doi: 10.2196/63572 (PMC11920652; doi:10.2196/63572)
Supplement: Multimedia Appendix 4 [file formative_v9i1e63572_app4.docx]

**List of translations of focus group and interview quotes.**

| Page | English Translation | German Original |
| --- | --- | --- |
| 12 | “And when it rings, we know, this woman is sitting at the edge of the bed. Then we know… We have ideas. Then we know how we have to organize the process or how to do the plan for the patient.” (FG1, P3) | “Und wenn es klingelt, dann wissen wir, ja, ok, die Frau hat sich an die Bettkante gesetzt. Dann wissen wir / dann haben wir Ideen. Dann wissen wir, wie wir den Ablauf machen müssen oder den Plan für den Patienten machen muss.” (FG1, P3) |
| 12 | “Well. Especially regarding falls there was a certain safety, because the notification actually always sets off and you don’t have to permanently do visual controls and go to the rooms. This certainly creates a feeling of safety, to know ‘Okay, the patient is not in danger of falls or similar right now’. Well and for coverage of the positioning and in order to see: ‘Okay, was there sufficient positioning? Have I supported the patient sufficiently? – it gives a certain feeling of safety when you can check this on the monitor” (FG2, P3) | „Schon. Also grad gegenüber Stürzen hat man schon ne gewisse Sicherheit, weil ja die Meldung eigentlich auch immer kommt und man nicht permanent ähm Blickkontakt zum Beispiel haben muss oder regelmäßig in's Zimmer gehen muss. Ähm, da ist auf jeden Fall n' Gefühl von Sicherheit da, dass man weiß "Okay, Patient ist da jetzt momentan nicht in Gefahr in Bezug auf Stürze oder Ähnlichem". Ähm, ja. Und einfach au zur Absicherung, klar, bei der Lagerung zum Schauen "Okay, war jetzt ne ausreichende Lagerung da? Hab ich die Patienten ausreichend beim Lagern überstü- äh unterstützt?", ist au ne gewisse Sicherheit, wenn man das am Monitor erkennt“ (FG 2, Person C) |
| 12 | “In nightshifts [With the IRMS] you immediately notice: Do people move, don’t they move. And before, okay you walk there every 2 to 3 hours, turn the people from left to right, have to wake them up and this is bad, when people are fast asleep and you see: Oh, he’s moving, moving sufficiently, perfect. AND, it’s very good that when somebody really has developed a decubitus here, we can prove: Okay, every 2 hours, the monitor has recorded it and there were immediate reactions, there were positionings and so we can demonstrate this.” (Interview) | „Weil auch in der Nacht, im Nachtdienst, es, ähm, man kriegt halt gleich mit: tun sich die Leute bewegen, oder tun sich nicht bewegen. Und davor wurde ja, okay du gehst alle 2-3 Stunden durch, tust die Leute von links nach rechts, musst sie aufwecken das ist ja auch "bleed", wenn die Leute tief und fest schlafen und so siehst du: Ahja, der bewegt sich, er bewegt sich ausreichend, perfekt. UND, das positive hier ist noch, wenn jemand wirklich einen Dekubitus jetzt gekriegt hat, wir haben dann den Nachweis: Okay, alle 2 Stunden, der Monitor hat es auf- aufgenommen und es wurde auch gleich gehandelt, es wurde gelagert und somit haben wir auch was vorzuzeigen.“ (Interview) |
| 14 | “Those who are hyperactive at night, you pick that up anyways, because then you are continuously close to the bed, because they’re ringing all the time, calling or doing anything else.” (FG2, P1). | “Aber die, die nachts da so hyperaktiv [sind], das kriegst ja eh mit, weil da stehst du sowieso dauernd am Bett, weil die da laufend klingeln, rufen oder sonst irgendwas tun.” (FG 2, Person A) |
| 14 | “This one patient, who always said: Ha, the spy betrayed me again, actually I wanted to try to go to the toilet by myself.’ There you stand next to them and say: ‘You shouldn’t walk alone; you are still quite week-kneed with the rollator.” (Interview) | „diese eine Patientin, wo immer gesagt hat: ha, der Spion hat mich wieder verraten, jetzt wollte ich es mal allein auf Toilette probieren. Dann stehst Du halt daneben und sagst: (verstellt die Stimme)"Sie solle net alleine laufe, Sie sin noch ziemlich wackelig am Rollator." (Interview) |
| 15 | “P3: I think they were not really aware, that there is a special mat in their bed. They didn’t really notice it, most of them. The others quickly mentioned it. But they didn’t mind either, whether there was something like that or not. So.  P1: Except for the ones who ranted, because they were worried, well – one once even said: “Am I being controlled”?  P3: Okay no, I’ve never come across that.  P1: And then there were sooooome, who worried the space for their cell phone plug. […]” (FG 2, P1, P3). | P3:  Ich glaub, das war denen gar nicht so bewusst, dass da jetzt nochmal ne Extramatte drin isch. Die haben das eigentlich relativ wenig wahrgenommen, die meisten. Die anderen haben‘s kurz angesprochen. Denen war das aber auch relativ egal, ob da jetzt sowas da ist oder nicht. Also  P2:  Bis auf die, die geschimpft haben, weil sie Sorge hatten, äh- Einer hat sogar mal geäußert: "Werde ich jetzt hier kontrolliert?".  P3:  Okay, nein, das ist mir jetzt noch [ nie  ] untergekommen.  P1: Und dann gab‘s nochmal ‘n paaaaar, die Sorge hatten, dass sie jetzt kein Platz mehr für ‘n Handystecker haben.“ |
| 16f. | Moderator: And if there was no MoMo starting from tomorrow on. How would you like that?  “Person 3: well…  Several: Laughing  Person 2: I would miss it. I would like to  Person 3: Yes, it would be missed. But the work still could be done. That’s not the way it is.  Person 2: Well maybe a thing or two would be more complicated again. Because we would have to do more visual controls at the patients’ beds, for patients who could wander or patients with dementia.  Person 3: Yes.  Person 1: Exactly, probably one fall or two would happen (2,5s)  Person 3: but those could not be prevented with MoMo, either.  Person 1: Yes, sure! We would certainly go back to text-book, which means positioning according to the clock, but-  Person 2: This would not make our work impossible  Person 1: (laughing) Exactly. Nursing can do that” (FG2, P1, P2, P3) | „Moderator: Und wenn ihr den MoMo jetzt morgen gar nicht mehr hättet: Was- Wie fändet ihr das?(5)  Person C?: Mm bjoaaa  Moderator*in B: <<lacht>>  Mehrere: <<lachen>> [ <<lachen>> ]  Person B: [Mir wird der schon fehlen.] Ich würd da schon gerne [ (???????????) Person C: [Jaa, e- es würd fehlen, aber (.) aber] die Arbeit wär wär trotzdem (.) weiterhin (1) möglich. So ist es nicht, ja.  Person B: Also würd vielleicht das ein oder andere tatsächlich wieder erschweren, [(?weil mehr /wieder?)] (??) Sichtkontrollen bei den Patienten, die dann aufstehen oder dementen Patienten  Person C: [ Jaa. ]  Person A: Genau, es würde (.) vielleicht einfach (1) zu dem ein oder anderen Sturz tatsächlich kommen, (2.5)  [Person C: Die sind aber teilweise auch nicht mit [MoMo (1) vermeidbar.]  Person A: [Man würde sicherlich (.)] Ja, ja, klar! Man würde sicherlich dazu übergehen, dann wieder eben nach Schema F, das heißt, nach Uhrzeit alle zu positionieren, (2) aber bfff (.) [ (.) Pflege-]  Person B: [Das würde unsere Ar]beit nicht unmöglich machen eben.  Person A: Ne, ge[nau,] Pflege kann das, ja, also <<lacht>>[ <<lacht>> (FG 2, several) |
